# Giant Bilateral Adrenal Myelolipoma with Congenital Adrenal Hyperplasia

**DOI:** 10.1155/2014/728198

**Published:** 2014-07-16

**Authors:** S. Al-Bahri, A. Tariq, B. Lowentritt, D. V. Nasrallah

**Affiliations:** ^1^Department of General Surgery, MedStar Union Memorial Hospital, Suite 655 B, Johnston Professional Building, 201 East University Parkway, Baltimore, MD 21218, USA; ^2^School of Medicine, Saba University, 27 Jackson Road, Suite 301, Devens, MA 01434, USA

## Abstract

Myelolipomas are rare and benign neoplasms, predominant of the adrenal glands, consisting of adipose and mature hematopoietic tissue, commonly discovered incidentally with increased use of radiologic imaging. Few cases of giant bilateral adrenal masses are reported, especially in the setting of congenital adrenal hyperplasia (CAH). We report the case of a 39-year-old male with a history of CAH secondary to 21-*α* hydroxylase deficiency on steroids since childhood, self-discontinued during adolescence, presenting with abdominal distension, fatigue, decreased libido, and easy bruising. Imaging revealed giant bilateral adrenal masses. He subsequently underwent bilateral adrenalectomy found to be myelolipomas measuring 30 × 25 × 20 cm on the left and weighing 4.1 kg and 25 × 20 × 13 cm on the right and weighing 2.7 kg. Adrenal myelolipomas are found to coexist with many other conditions such as Cushing's syndrome, Addison's disease, and CAH. We discuss the association with high adrenocorticotropic hormone (ACTH) states and review the studies involving ACTH as proponent leading to myelolipomas. Massive growth of these tumors, as in our case, can produce compression and hemorrhagic symptoms. We believe it is possible that self-discontinuation of steroids, in the setting of CAH, may have resulted in the growth of his adrenal masses.

## 1. Introduction

Although benign and endocrinologically inactive, myelolipomas are rare tumors of the adrenal gland that still present to surgeon's clinic for further evaluation. They consist of adipose and hematopoietic tissue and have been increasingly discovered due to the increased use of imaging such as computed tomography and magnetic resonance imaging for other medical conditions. Their incidence has been reported to be less than 0.4%, with a few growing to the size of giant bilateral adrenal masses. The development of these tumors has been associated with congenital adrenal hyperplasia (CAH) as in this case and with other adrenal hormonal diseases, which will be the focus of this discussion.

## 2. Case Presentation

**Figure 1:**
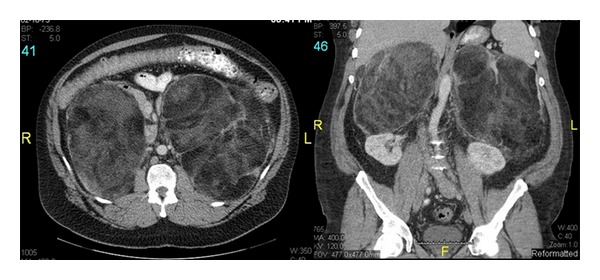
Axial and coronal views of the bilateral adrenal masses on computed tomography imaging showing a portion of the left adrenal mass interposed between the aorta and left adrenal vein bowing the vein anteriorly. Both masses are composed of broad sheets of fat, delicate septations, and isodense material. The bowel is displaced anteriorly and inferiorly but is showing normal bowel gas pattern.

A 39-year-old male with a history of congenital adrenal hyperplasia, diagnosed with 21-*α* hydroxylase deficiency as a child, presented to the clinic with fatigue, headaches, decreased libido, easy bruising, and increasing abdominal distension. It was not clear from the history how he was diagnosed at birth, but the patient had been on steroids throughout his childhood but had self-discontinued them during adolescence due to concerns of weight gain. Imaging done as part of his adrenal pathology workup revealed bilateral adrenal masses measuring 16 × 12 × 15 cm on the right and 20 × 20 × 25 cm on the left one year prior to presentation to surgeon's clinic. The patient was then restarted on steroids and underwent a repeat computed tomography (CT) scan one year later, which showed interval increase in the adrenal masses (Figure 1). No comment was made by the radiologist on the normal remnant adrenal tissue, as there was no clear plane separating his normal adrenal tissue from the adrenal masses. Surgical consultation was then requested as the repeat imaging study showed compression of the masses against his kidneys and bowels but no sign of renal or intestinal obstruction. His only abdominal symptom that remained was increasing abdominal distension and discomfort. He was thus scheduled for bilateral adrenalectomy after multispecialty consultation with his endocrinologist, urologist, and general surgeon.

As part of his preoperative workup, his testosterone level was 506 ng/dL (normal 241–827), 17-hydroxy-progesterone was 14, 076 ng/dL (42–196), and ACTH level was 42 pg/mL (6–50). He had been restarted on hydrocortisone 10 mg by mouth twice daily and fludrocortisone acetate 0.1 mg by mouth daily by his endocrinologist for a year prior to presentation.Utilizing a midline incision, a Maddox maneuver was performed, releasing the splenic flexure and descending colon inferiorly to the sigmoid colon. Using blunt dissection, the left-sided adrenal mass was easily freed from the kidney, which was identified at its inferomedial aspect. The left adrenal vein was found, clipped, and divided. The left adrenal mass was freed from its surrounding attachments using sharp dissection. This was followed by a Cattel-Brach maneuver, which freed the ascending colon up to the hepatic flexure as well as the gastrohepatic ligament. A Kocher maneuver was then performed which freed the duodenum and allowed better access to the right-sided adrenal mass in the retroperitoneum. The right adrenal mass was then released from its surrounding attachments by elevating the liver and retracting the right colon medially, as well as a combination of sharp and blunt dissection. The right adrenal vein was identified coursing into the inferior vena cava and divided using an endovascular stapler. The mass was then removed and both fossae were evaluated for bleeding. Attempts were made throughout the operation to identify a plane separating the masses from normal adrenal tissue, but due to the sheer size of these masses, no such plane could be found and thus the entire adrenal glands were removed. All other organs were inspected and found to be normal, and two drains were placed to drain the retroperitoneal fossae bilaterally and the abdomen was closed.

**Figure 2:**
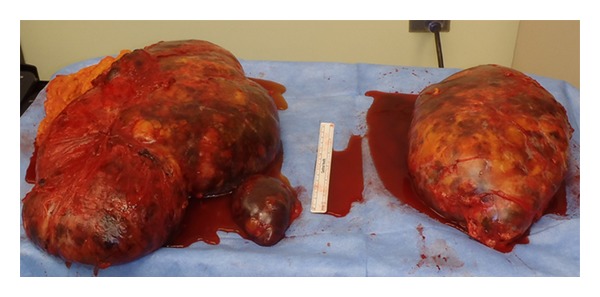
Gross pathologic specimen of the bilateral adrenal masses was described by the pathologist as left adrenal gland myelolipoma weighing 4116 g and measuring 30 × 25 × 20 cm, and right adrenal gland myelolipoma weighing 2672 g and measuring 25 × 20 × 13 cm, both exhibiting hemorrhage, hemosiderin deposition, fibrosis, and fat necrosis, but no sign of malignancy.

Postoperatively, the patient was transferred to the intensive care unit as a precaution to monitor for an Addisonian crisis. His postoperative course was marked by persistent tachycardia but appropriate blood pressure. He was started on the appropriate steroid dosage, and supportive care with fluids was continued. A CT scan of the chest was done in the postoperative period to rule out pulmonary embolism due to persistent sinus tachycardia unresponsive to fluids or steroids. The CT scan was requested by the cardiologist involved in the case and was negative for pulmonary emboli showing only a small left-sided pleural effusion. The tachycardia resolved upon return of bowel function and the patient was transitioned to medications by mouth. The drains were removed by postoperative day 7 as the output decreased. The patient was discharged on postoperative day 8. The final pathology showed bilateral benign myelolipoma measuring 30 × 25 × 20 cm on the left and weighing 4.1 kg and 25 × 20 × 13 cm on the right and weighing 2.7 kg (Figure 2).

Since surgery, he has followed up every 6 months and has complained of incisional pain but no other abdominal symptoms. The pain responded to an intercostal nerve block, which was done by his pain specialist. His postoperative hormone levels while continuing on his preoperative steroid medications and dosage showed an initial drop in testosterone levels to 34 ng/dL gradually returning to 227 ng/dL (241–827) on his next visit. He was not interested in testosterone replacement and does not wish to have any children in the future. His 17-hydroxy-progesterone level did normalize to 175 ng/dL (42–196), and his ACTH level was 46 pg/mL (6–50).

## 3. Discussion

Myelolipoma is a rare, benign, and endocrinologically inactive neoplasm. It is predominantly found in the adrenal glands and consists of adipocytes and mature hematopoietic tissue. It was first described in 1905 by Gierke as a mass lesion that is composed of mature fat tissue and mixed myeloid and erythroid tissue [1]. Subsequently, Oberling named it “formations myelolipomatoses” in 1929 [2]. In the past, this tumor was primarily detected on autopsies, by symptoms due to compression on surrounding viscera, or by altered hormonal production by the ipsilateral adrenal gland. Lately, due to widespread use of radiological studies such as ultrasonography, CT, and magnetic resonance imaging (MRI), incidental discovery of indolent adrenal myelolipomas has become more common [3]. Due to their high fat content, they have a very characteristic appearance on imaging studies. They appear echogenic on ultrasound, low attenuated lesions on CT, and hyperintense on T1-weighted in-phase MRIs [4]. The reported incidence of adrenal myelolipoma ranges from 0.08 to 0.4% and constitutes 15% of all adrenal incidental masses. This tumor affects both genders equally and is commonly found in the fifth to seventh decade [5].

In the majority of the cases, adrenal myelolipomas are unilateral and rarely exceed 4 cm. However, very large and bilateral myelolipomas have been reported. Tumors exceeding 8 cm are referred to as giant myelolipomas [6]. The largest unilateral adrenal myelolipoma reported in the literature weighed 6 kg and measured 31 × 24.5 × 11.5 cm [7]. Very few studies in the past have reported bilateral adrenal myelolipomas and we present one of these rare entities. The largest bilateral adrenal myelolipoma reported weighed 5.8 kg (23 × 11 × 19 cm) on the left and 0.78 kg (15 × 13 × 6.8 cm) on the right [8]. In the case of bilateral neoplasms, left-sided masses are usually larger than the right. It has been speculated that the asymmetric growth of these myelolipomas is due to the space limiting constraints by the liver on the right side and unrestricted growth on the left side [9].

Massive growth of this neoplasm can produce symptoms such as flank pain and abdominal discomfort due to rupture, hemorrhage, or necrosis [10]. More severe symptoms include hematuria, renovascular hypertension, and even surgical emergencies such as retroperitoneal hemorrhage [6, 11]. Therefore, it is important to realize the significance of incidental findings and management should be geared towards preventing such severe consequences. Furthermore, due to the recent increase in the number of incidentally found adrenal myelolipomas, firm guidelines are now needed to help decide between watchful waiting versus surgical removal of these benign tumors. Recommendations suggest that asymptomatic myelolipomas of less than 4 cm should be monitored expectantly with annual CT scans. Since the risk of spontaneous rupture or bleeding is minimal in these small myelolipomas, observation can avoid lifelong steroid substitution [6, 12]. Surgery is indicated if the tumor exceeds 7 cm because of increased risk of spontaneous rupture and retroperitoneal hemorrhage [10, 12]. Some surgeons recommend resection when tumor size is greater than 5-6 cm since potentially malignant tumors of the adrenal gland also present as nonfunctional tumors [13]. Others recommend surgical removal of myelolipomas greater than 4 cm [5, 14] because of the rare chance of rupture and other associated complications. In the past, tumor size of more than 5-6 cm was considered a contraindication for laparoscopic resection. However, recent studies have shown that it is a safe approach with outcomes comparable to an open procedure [6, 15]. In the case of bilateral myelolipomas, recommendations suggest removal of the larger and more symptomatic side and observation of the contralateral side for as long as possible to avoid lifelong supplementation of steroids [12]. The literature also demonstrates that myelolipomas can grow significantly during the observation period and a few studies have reported spontaneous hemorrhage with minor trauma in these patients [16, 17]. In our case, a bilateral adrenalectomy was performed because bilateral giant myelolipomas were present in our patient with congenital adrenal hyperplasia (CAH), with both masses growing significantly over a period of one year. Surgical resection is advised by most, regardless of the size of the myelolipoma, if the patient is symptomatic, or if malignancy could not be ruled out [5, 10, 12].

Although myelolipoma was first reported 100 years ago, its tumorigenesis still remains a mystery. There are many barriers that prevent detailed investigation of its etiology. The limited incidence of this neoplasm leads to highly inconclusive experiments. In addition, its unique mixture of fat and mature hematopoietic cells makes establishment of cell lines almost impossible limiting in vitro studies [18]. Finally, its low incidence makes paraffinization of surgical specimens a necessity leading to difficulty in performing retrospective molecular or functional analysis [18]. Nonetheless, several historical hypotheses of adrenal myelolipoma formation exist. They include its derivation from bone marrow emboli lodging in the adrenal gland and its origin from embryonic primitive mesenchymal cells [10, 18, 19]. The most favorable theory is of metaplastic transformation of undifferentiated stromal cells leading to myelolipoma formation [12, 18, 20], which will be discussed further.

Adrenal myelolipomas are found to occur concurrently with other conditions with hormonal dysfunction such as cortical adenoma [21], ganglioneuroma [22], carcinoma [23], pheochromocytoma [24], Cushing's and Conn's syndrome [25], overproduction of dehydroepiandrosterone sulphate (DHEAS), and CAH due to 21 hydroxylase deficiency or 17 *α*-hydroxylase deficiency [26, 27]. Among these, the two most common causes are CAH and Cushing's syndrome [6]. There are different hypotheses presented in the literature that describe the etiology and pathogenesis of adrenal myelolipomas. One of the prevalent beliefs implies that chronic adrenocorticotropic hormone (ACTH) stimulation causes metaplasia of adrenocortical cells leading to myelolipomas [13, 28, 29]. The development of myelolipomas in patients with excessive secretion of ACTH such as in CAH [10, 30, 31], Nelson's syndrome [32], and Addison's disease [33] also supports this theory. Most of these reported patients were untreated and were exposed to long periods of elevated ACTH. Adrenal myelolipomas are also found to be associated with chronic stressful conditions such as diabetes mellitus, hypertension, obesity, chronic inflammatory processes, and malignancy [34, 35]. In addition, myeloid metaplasia in the adrenal cortex is also observed in patients with severe burns or cancer who are under long periods of intense stress [36]. Another study that supports the theory of ACTH inducing adrenal myelolipomas describes the occurrence of adrenal myelolipoma in a patient with ectopic ACTH producing oat cell lung carcinoma [37]. On the other hand, there have been studies that demonstrate a limited role of ACTH in the development of myelolipomas. ACTH-independent Cushing's syndrome has also been shown to be associated with myelolipomas [38]. In addition, histological analysis in one study revealed that myelolipoma does not overexpress ACTH receptors as expected by the above theories [39].

## 4. Conclusion

After reviewing the literature, we have found that there is no definite consensus on surgical management of adrenal myelolipomas. Although hormonally inactive, we discussed the possibility of accelerated growth of these tumors in the setting of congenital adrenal hyperplasia. Although the patient showed no signs of obstruction at the time of presentation, we felt that a discussion with the patient about surgical resection was warranted due to his mild discomfort and distension. This is especially the case in the setting of a young and relatively healthy patient undergoing an elective procedure, as opposed to a more urgent operation secondary to obstruction from progressively enlarging masses.
